# Dimerization of Acetic Acid in the Gas Phase—NMR Experiments and Quantum-Chemical Calculations

**DOI:** 10.3390/molecules25092150

**Published:** 2020-05-04

**Authors:** Ondřej Socha, Martin Dračínský

**Affiliations:** 1 Institute of Organic Chemistry and Biochemistry, Czech Academy of Sciences, Flemingovo nám. 2, 166 10 Prague, Czech Republic; ondrej.socha@uochb.cas.cz; 2 Faculty of Mathematics and Physics, Charles University, Ke Karlovu 3, 166 10 Prague, Czech Republic

**Keywords:** NMR spectroscopy, quantum-chemical calculations, phase transitions, carboxylic acids, hydrogen bonding

## Abstract

Due to the nature of the carboxylic group, acetic acid can serve as both a donor and acceptor of a hydrogen bond. Gaseous acetic acid is known to form cyclic dimers with two strong hydrogen bonds. However, trimeric and various oligomeric structures have also been hypothesized to exist in both the gas and liquid phases of acetic acid. In this work, a combination of gas-phase NMR experiments and advanced computational approaches were employed in order to validate the basic dimerization model of gaseous acetic acid. The gas-phase experiments performed in a glass tube revealed interactions of acetic acid with the glass surface. On the other hand, variable-temperature and variable-pressure NMR parameters obtained for acetic acid in a polymer insert provided thermodynamic parameters that were in excellent agreement with the MP2 (the second order Møller–Plesset perturbation theory) and CCSD(T) (coupled cluster with single, double and perturbative triple excitation) calculations based on the basic dimerization model. A slight disparity between the theoretical dimerization model and the experimental data was revealed only at low temperatures. This observation might indicate the presence of other, entropically disfavored, supramolecular structures at low temperatures.

## 1. Introduction

Noncovalent interactions play a significant role in many chemical and biological processes. For instance, molecular recognition, protein folding, and crystal packing are phenomena where these interactions shape the structure, properties, and function of molecules and materials. Hydrogen bonding is the most important noncovalent interaction. Hydrogen bonds are typically an order of magnitude weaker than covalent bonds, and yet they determine, for example, the secondary structure of large biopolymers such as DNA or proteins.

Carboxylic acids possess a functional group that can simultaneously serve as a hydrogen-bond donor and acceptor. Therefore, carboxylic acids can form a number of supramolecular structures, and they are often used as model systems to gain an insight into the hydrogen-bonding interaction [1]. Acetic acid, which is an archetypal carboxylic acid, serves as an excellent example to demonstrate this. 

Crystalline acetic acid is comprised of linear chains of hydrogen-bonded molecules [2]. In the gas phase, on the other hand, the presence of centrosymmetric cyclic dimers of acetic acid has been confirmed by several experimental studies [3,4,5,6]. Each of the dimers contains two equivalent strong O–H···O=C hydrogen bonds. The dimerization enthalpy and entropy have been estimated to be about −15 kcal/mol and −37 cal/K·mol, respectively, by several experiments, such as IR experiments and vapor-density or thermal-conductivity measurements [7]. However, NMR spectroscopy experiments of gaseous acetic acid have estimated the dimerization enthalpy and entropy as about −18 kcal/mol and −42 cal/K·mol, respectively [8]. There is no clear consensus about the prevailing supramolecular structure in liquid acetic acid [9]. While neutron diffraction [10] and time-domain Raman spectroscopy [11,12] experiments have suggested that liquid acetic acid primarily consists of cyclic dimers, other experiments, such as large-angle X-ray scattering [13] and IR [14] and Raman [15] spectroscopy, have suggested that the main structural patterns in liquid acetic acid are linear chains similar to those found in the solid. Furthermore, other studies have indicated that acetic acid monomers and cyclic trimers coexist with the dimers and chains in the liquid phase [16,17].

Acetic acid and its supramolecular complexes have also been investigated in many theoretical studies [18,19,20,21,22]. Thanks to its small size, acetic acid can be used for benchmarking different computational approaches including various density-functional-theory (DFT) functionals or post-Hartree–Fock methods [23,24,25]. Even expensive methods, such as coupled clusters (CC) and quadratic configuration interaction, are feasible [26]. Besides the estimation of binding energies, quantum-chemical calculations have been utilized for predictions of NMR, IR, and Raman spectroscopic parameters [27,28].

In this work, we combined NMR experiments with advanced computational approaches to better understand the hydrogen-bonding interactions of acetic acid. The experimental part of this study aimed to precisely measure the thermodynamic parameters of acetic acid dimerization using gas-phase NMR spectrometry and to explain the previously observed discrepancies of NMR-derived dimerization enthalpy and entropy with those obtained by other experiments. The goal of the computational part was to accurately calculate the dimerization Gibbs energy and proton chemical shifts of the monomeric and dimeric forms of acetic acid in the gas phase. Several DFT and post-Hartree–Fock methods were employed for energy and NMR computations. In addition, we utilized path-integral molecular dynamics (PIMD) simulations to obtain an insight into nuclear quantum effects such as nuclear delocalization and proton tunneling in the acetic acid dimer. A comparison of theoretical and experimental thermodynamic and spectroscopic parameters was used to discuss the accuracy of the computational methods, as well as to provide a validation of the basic dimerization model of gaseous acetic acid.

## 2. Models and Methods

### 2.1. Theoretical Model

A simple dimerization model was assumed:

(1) 2M↔D

where M and D stand for the monomer and dimer of acetic acid, respectively. The equilibrium constant *K* is then defined in terms of partial pressures:

(2) $$K=\frac{p_{D}}{p_{M}^{2}}.$$

According to the Dalton law of partial pressures, the total pressure *p_tot_* can be obtained using the formula:

(3) $$p_{tot}=p_{M}+2p_{D}.$$

The observed chemical shift *δ* is calculated as a weighted average of monomeric and dimeric shifts *δ_M_* and *δ_D_*, respectively:

(4) $$\delta =\frac{p_{M}}{p_{tot}}\;\left(\delta_{M}-\delta_{D}\right)+\delta_{D}$$

where the partial pressure of the monomeric form *p_M_* is derived using the above equations, which leads to the expression:

(5) $$p_{M}=\frac{\sqrt{1+8Kp_{tot}}-1}{4K}$$

The equilibrium constant *K* is directly related to the change of enthalpy ∆*H* and entropy ∆*S* of dimerization through the van’t Hoff equation:

(6) $$K=\exp\left(-\frac{\Delta H-T\Delta S}{RT}\right)$$

A detailed derivation of the above formulae is available in the Supplementary Materials.

### 2.2. NMR Experiments

The experimental setup consisted of an evacuated torch-sealed glass insert (⌀4 mm; *L* = 40 mm) or a fluorinated ethylene propylene copolymer (FEP) insert sealed with a polytetrafluoroethylene (PTFE) stopper filled with acetic acid vapors. The glass insert was evacuated and heated above 150 °C to remove any residual moisture prior to the acetic acid injection (still under reduced pressure). The insert was fitted into a regular ⌀5 mm NMR tube. The restricted length of the insert confined the sample within the NMR detection coil in the probe and limited heat transfer to the surrounding space. This assembly enabled a regular 20 Hz rotation for *B*_0_ (the spectrometer magnetic field) inhomogeneity averaging. Automatic gradient shimming procedures were inapplicable due to the high diffusivity and low concentration of the vapors, and there was no lock signal. These conditions led to slightly broadened signals despite the effort to manually adjust the magnetic-field homogeneity. This, however, introduced only a negligible error to the chemical-shift measurement. No chemical-shift reference was added to the sample, and only the differences *∆δ = δ_OH_ − δ_Me_* are reported, where *δ_OH_* and *δ_Me_* are the proton chemical shifts of the OH and CH_3_ groups, respectively. We did not use any internal chemical-shift standard because the magnetic susceptibility of liquid acetic acid is substantially different from that of acetic acid vapors. Therefore, the resonance frequency of a standard would also have differed in the solution and in the vapor, and we would have observed two different signals of the standard (solution and gas phase). Furthermore, tetramethylsilane (TMS), the most common NMR standard, is very volatile, and it would probably not have persisted in the acetic acid solution under reduced pressure.

The measurements were performed on a 500 MHz Bruker Avance II NMR spectrometer (Bruker BioSpin, Germany) equipped with a TBO probe, allowing for measurement in a wide temperature range from −150 to 150 °C. The temperature was regulated using a sensor and a heater inside the probe at a constant air flow rate of 535 l/h. The temperature sensor was calibrated using ethylene glycol prior to the experiment.

Temperature series of the ^1^H–NMR spectra of acetic acid in the glass insert were measured from 150 to 30 °C, with a 5 °C step and a 15 min equilibration period between each step. The spectra were acquired with 1024 transients, 9012 points in the time domain, a spectral width of 18 ppm, a repetition time of 0.3 s, and an acquisition time of approximately 14 min per temperature point. In total, three such datasets with different sample concentrations were measured. The spectra in the FEP inserts were acquired as pseudo-2D time series with the acquisition time of 2.5 min per spectrum, 128 transients, 12,288 points of free induction decay (FID), a spectral width of 25 ppm, and a repetition time of 0.6 s. Measurements were performed at four different temperatures (25, 60, 80, and 100 °C).

Water content in the samples was estimated by the following procedure. Acetic acid used for sample preparation in our experiments was delivered from the manufacturer in 1.5 mL glass ampules. Water content was determined using standard liquid NMR measurement based on the following information: Pure acetic acid solidifies from the solution (acetic acid–water) at temperatures under the melting temperature of acetic acid. However, a mixture of remaining acetic acid and water stays liquid above –27 °C, which is the eutectic point of the mixture. An NMR tube filled with 0.5 mL of acetic acid was cooled down below the freezing point of acetic acid. In Figure S1, two NMR spectra at –6 °C are shown. One spectrum directly corresponds to the acetic acid from the ampule, and the other one is with the addition of 7 mol% of water. It is clear from the comparison of these two spectra that there was more than ten times less water in the original sample. Therefore, we concluded that the water content in the acetic acid used in our experiments was far below 1 mol%.

#### 2.2.1. Pressure Estimation

In the gas-phase measurement, the actual pressure in the NMR tube must be known for the calculation of thermodynamic quantities. It was not a straightforward task to obtain an accurate pressure value because the sample was either in a torch-sealed tube or in a leaky polymeric insert, which means that any pressure setting was lost during or after the tube sealing. Therefore, the pressure was estimated from the condensation temperature *T*_0_. Generally, when condensation occurs, a second set of NMR signals starts to appear in the spectrum. In the case of acetic acid and other diamagnetic liquids, these signals are shifted to lower Larmor frequencies than the signals from the gas phase as a result of the negative magnetic susceptibility of the bulk material. It is thus easy to distinguish between gas- and liquid-state signals. At this point, the pressure *p*_0_ can be determined based on the temperature *T*_0_ and the a priori knowledge of the temperature dependence of given liquid-vapor pressure. The vapor pressure is tabulated for many materials in terms of Antoine equation coefficients [29,30]; see the Supplementary Materials. In the case of airtight glass inserts under the assumption of ideal gas behavior, the pressure only depends on the temperature according to the expression:

(7) $$p_{tot}\left(T\right)=\frac{p_{0}}{T_{0}}$$

Measurements in the FEP inserts were performed at constant temperature. The FEP inserts were not airtight, and the acetic acid vapors continuously leaked from the insert for several hours or days, depending on the temperature. After the evaporation of the liquid, the partial pressure of the acetic acid showed an exponential decay during the time *t*, which was best observed on the integral intensities *I*(*t*) of the methyl-group NMR signal. The pressure at a given time (snapshot) was calculated using the formula:

(8) $$p_{tot}\left(t\right)=\frac{I\left(t\right)}{I\left(t\right){|}_{t=t_{0}}}p_{0}$$

where the time *t*_0_ is tied to the beginning of the pressure decay.

#### 2.2.2. Data Processing

Temperature and pressure series of the spectra were acquired and processed using the TopSpin 3.5 software (Bruker). An exponential apodization of 5 Hz was applied to improve the signal-to-noise ratio, and the baseline was corrected using a 5th-order polynomial. Data were zero-filled to 3,2768 points. Chemical shifts and line widths were obtained through fitting Lorentzian line shapes into the datasets. The temperature or pressure dependencies of chemical shifts were subsequently fitted using Expression (2), yielding the thermodynamic parameters ∆*H* and ∆*S*. Datasets from glass-insert measurement were treated separately; the optimized parameters of ∆*H*, ∆*S*, and chemical-shift differences (*δ_OH_ − δ_Me_*) of the monomer ∆*δ_M_* and dimer ∆*δ_D_* are available in the Supplementary Materials. The global fitting procedure was employed for FEP measurement. The parameters ∆*H* and ∆*S* were shared between datasets, while ∆*δ_D_* and *p_corr_* were independently optimized for each dataset. The parameter *p_corr_* was subtracted from the pressure in order to take into account the small baseline distortion that leads to incorrect pressure behavior $\underset{t\to \infty }{\lim}p_{tot}\left(t\right)\neq 0$:

(9) $$p_{tot}\left(t\right)=\frac{I\left(t\right)}{I\left(t\right){|}_{t=t_{0}}}p_{0}-p_{corr}$$

The value of *p_corr_* was always below 0.005 atm.

The chemical shift of the monomer ∆*δ_M_* was fixed at the value of 3.76 ppm, which was obtained through extrapolation to zero pressure from the measurement at 150 °C. To improve the convergence of the optimization procedure, the Savitzky–Golay smoothing filter of the 3rd order with a 21-point window was applied to the pressure data *p_tot_*(*t*). Further window widening had only a negligible effect on the optimized parameters. There were between 600 and 1200 pressure points in each dataset.

The uncertainty of the optimized parameters was estimated from the *p*_0_ pressure uncertainty. Two contributions to *p*_0_ uncertainty were considered: systematic contribution (10% of *p*_0_) and dataset-specific contribution (5% of *p*_0_). Both originated from the uncertainty of the point where the liquid evaporated, see below. Though these contributions could be merged into one, they tend to behave differently in terms of error propagation. The systematic error seems to have had only a minor influence on the uncertainties of the optimized thermodynamic parameters. The main source of the error was thus a slight inconsistency in *p*_0_ determination between individual data sets. Standard deviations of the fitted parameters were estimated using the Monte Carlo method. In 1000 iterations, the predictors *p*_0_ were re-sampled from normal distributions with variance parameters reflecting the above uncertainties. The set of re-optimized parameters was statistically analyzed, yielding standard deviations of all optimized parameters and ∆*H* and ∆*S* covariance. All fitted parameters including their uncertainties are in the Supplementary Materials.

### 2.3. Computational Methods

Energy calculations, geometry optimization, and the computation of NMR parameters were carried out utilizing the Gaussian16 software package [31]. For all of these calculations, the “frozen-core” approximation was employed. The B3LYP [32,33] calculations used an “ultrafine” integration grid, and the effect of semiempirical Grimme’s dispersion correction (GD3) [34] on the structure and the binding energy of acetic acid was evaluated. Energy computations were performed with Dunning’s augmented correlation-consistent double-*ζ* (Aug-cc-pVDZ) to quintuple-*ζ* (Aug-cc-pV5Z) basis sets [35,36]. The basis set superposition error (BSSE) was estimated using the counterpoise method (CP) [37]. C2 symmetry was imposed for all calculations of the acetic acid dimer.

All geometry optimizations were carried out with “tight” convergence criteria, and the optimized structures possessed no imaginary vibrational frequencies, with one exception. In the case of the dimer structure optimized using the second order Møller–Plesset perturbation theory (MP2) [38] with the Aug-cc-pVQZ basis set, the harmonic analysis could not be done because of excessive computational requirements. However, this structure was pre-optimized at the MP2/Aug-cc-pVTZ level, yielding no imaginary frequencies.

The input geometries for all MP2 and coupled cluster with single, double, and perturbative triple excitation (CCSD(T)) [39,40,41,42] energy and/or NMR computations were optimized at the MP2/Aug-cc-pVQZ level. To further improve the precision of MP2-calculated energies, the correlation energy *E_corr_* was extrapolated to the complete basis set (CBS) limit using Helgaker’s formula [43]:

(10) $$E_{corr}^{X}=E_{corr}^{CBS}+\frac{A}{X^{3}}$$

where *X* = 2,3,4,5 is the “cardinal number” of the basis set used to calculate the respective energy *E^X^* and *A* is the system-dependent free parameter. As can be seen in Figure S8, the double-*ζ* basis set yielded inconsistent results; therefore, these values were excluded from the extrapolation. The Hartree–Fock (HF) energy calculated with Aug-cc-pV5Z was then added to $E_{corr}^{CBS}$ in order to obtain the MP2/CBS binding energy $E_{MP2}^{CBS}$. More detailed values are in Table S3 in the Supplementary Materials. A different approach to CBS extrapolation was employed in the case of CCSD(T). Due to prohibitively demanding requirements of the CCSD(T) method, only a single-point energy calculation with the Aug-cc-pVTZ basis set was possible. However, it has been observed that the difference between the MP2 and CCSD(T) calculated energy is only slightly dependent on the basis set. Formula (4), which has been proposed to exploit this fact [44], was employed here:

(11) $$\Delta E_{CCSD\left(T\right)}^{CBS}=\Delta E_{MP2}^{CBS}+{\left(\Delta E_{CCSD\left(T\right)}-\Delta E_{MP2}\right)}_{\mathrm{smaller}\;\mathrm{basis}\;\mathrm{set}}$$

For all MP2 and CCSD(T) calculations, the entropy change ∆*S* and the thermal contributions to the enthalpy change ∆*H_thermal_* of dimerization were calculated at the MP2/Aug-cc-pVTZ level. These values were subsequently added to the MP2- and CCSD(T)-calculated single-point energy values ∆*E* according to the equation:

(12) $$\Delta H=\Delta E+{\left(\Delta H_{MP2}-\Delta E_{MP2}\right)}_{\mathrm{Aug}-\mathrm{cc}-\mathrm{pVTZ}}$$

The values of ∆*G*^0^ and ∆*H* were reported for direct comparison with experimental values. No special treatment of vibronic modes for the correction of entropy or enthalpy was applied.

For the B3LYP and MP2 calculations of ^1^H chemical shielding, the gauge-independent atomic orbital (GIAO) method [45] with the Aug-pcS-2 basis set [46] was used. These computations were performed on the structures optimized with the Aug-cc-pVQZ basis set using the same method as the subsequent NMR calculations.

PIMD simulations were performed by the CASTEP program [47], version 17.2, using an NVT ensemble, the temperature of 300 K, a Langevin thermostat, a 0.5-fs integration time step, ultrasoft pseudopotentials [48], and a planewave cutoff energy of 300 eV. The integrals were taken over the Brillouin zone using a Monkhorst–Pack [49] grid of the minimum k-point sampling of 0.1 Å^−1^. Electron-correlation effects were modeled using the generalized-gradient approximation of Perdew, Burke, and Ernzerhof [50]. The acetic acid monomer and dimer were placed in a cubic periodic box of 15 × 15 × 15 Å^−1^, and the atomic positions were optimized by energy minimization prior to the PIMD runs at the same computational level. No symmetry constraints were applied during the simulation of the dimer. The production runs of the monomer and dimer simulation of the lengths of 4 and 6 ps, respectively, were preceded by 2-ps equilibration. The path-integral propagation used a Trotter decomposition of all nuclei into 16 beads.

Time-averaged NMR parameters were computed from 1000 snapshots from the MD and PIMD simulations. The B3LYP functional with the Aug-pcS-2 basis set was employed for the calculation of NMR shieldings.

## 3. Results and Discussion

### 3.1. NMR Experiments

**Figure 1:**
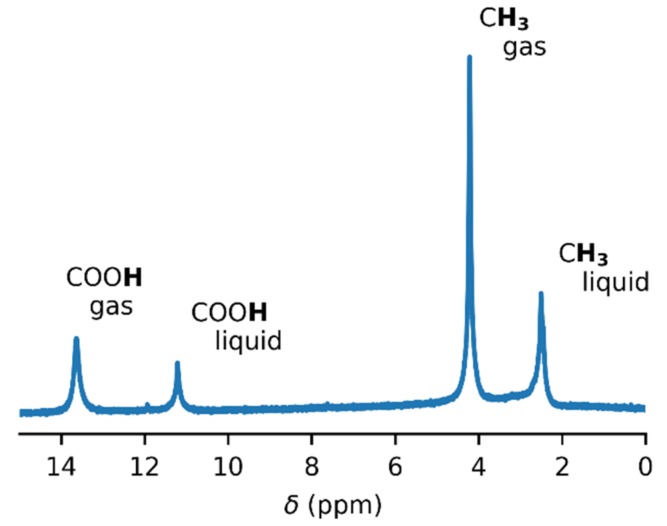
The ^1^H–NMR spectrum of acetic acid in a glass insert at the temperature of 60 °C. The spectrum was not referenced.

**Figure 2:**
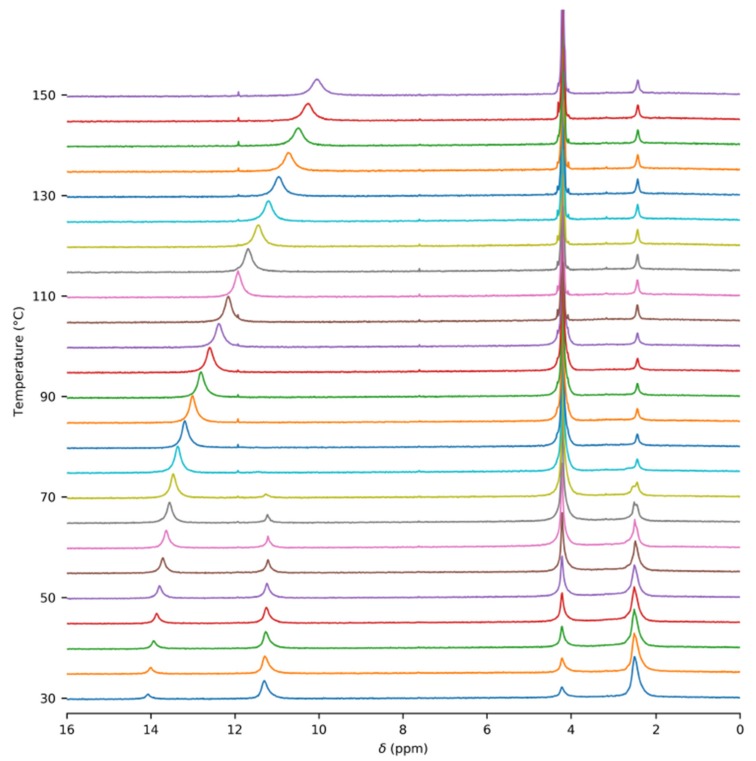
Temperature series of ^1^H–NMR spectra of acetic acid (c = 7:9 mM) in the glass insert. The spectra were not referenced.

In order to obtain accurate thermodynamic parameters of acetic acid complexation, we performed variable-temperature and variable-pressure NMR measurements. Initially, we carried out the experiments in glass inserts. These inserts were torch-sealed in order to ensure a constant amount of acetic acid throughout the measurements. Figure 1 shows an example of the ^1^H–NMR spectrum of acetic acid in a glass insert at the temperature of 60 °C. Two sets of signals were present, corresponding to the gas and liquid states of acetic acid. The temperature series of the ^1^H–NMR spectra of acetic acid in the glass insert are in Figure 2 and in the Supplementary Materials (Figures S4–S6). The signal of the methyl group in gaseous acetic acid was shifted from the signal of the liquid by about 1.7 ppm towards higher frequencies. This was the result of the different magnetic susceptibility of both environments. The diamagnetic nature of liquid acetic acid caused the shielding of all nuclei within, but there was no such effect in gases. In addition to that, a different intermolecular bonding environment led to further chemical-shift changes.

**Figure 3:**
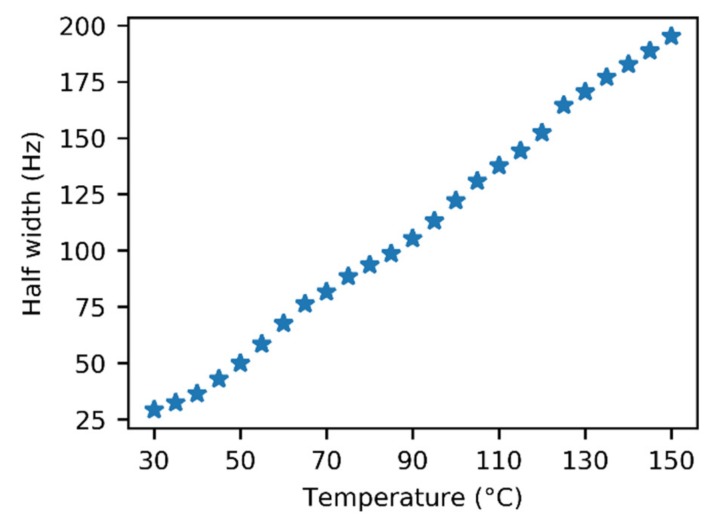
Temperature dependence of the half width of the ^1^H–NMR signal of the acetic acid COOH group in the glass insert.

An unexpected temperature-dependent line broadening of the COOH signal was observed in the spectra of gaseous acetic acid in the glass insert. Strangely, the broadening became more prominent as the temperature increased. This dependence is depicted in Figure 3 and is also noticeable in the spectra (Figure 2 and Figures S4–S6). Though some broadening can be expected in solution, where it can indicate a chemical exchange between various supramolecular structures, it is surprising in the gas phase. In our model, we assumed only one process, which was the dimerization of acetic acid monomers. We expected this process to have a relatively high exchange rate at the temperatures attained in our experiments, leading to narrow averaged signals. This broadening pointed to another unexpected exchange process. One of the possible explanations may be related to water presence in the sample. However, we did not expect a considerable interaction, such as hydrogen bonding between acetic acid and water molecules in the gas phase because of the high purity of the sample. Water content was determined to be far below 1% (molar) using liquid NMR (see the Methods and Supplementary Materials for more details). Moreover, we expected the interaction with water to be energetically less favorable than the binding to another acetic acid molecule. The only other explanation of the unexpected line broadening was the interaction of acetic acid molecules with the walls of the glass insert, specifically with the oxygen-containing functional groups on its surface. The existence of these interactions has already been indicated in a previous study [51].

The temperature dependence of the chemical-shift difference *∆δ = δ_OH_ − δ_Me_* was further analyzed. The basic dimerization Model (2) was fitted to the experimental data. The optimized parameters (∆*δ_M_,* ∆*δ_D_,* ∆*H* and ∆*S*) showed a strong dependence on the pressure *p_tot_*, despite the fact that they were expected to be pressure-independent. This supported the hypothesis of the presence of another chemical exchange with molecules adsorbed on the glass walls.

**Figure 4:**
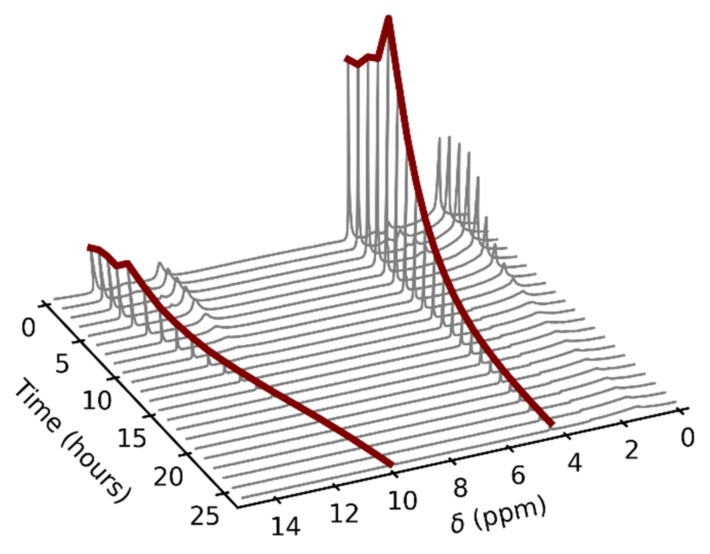
An example of the time dependence of the acetic acid ^1^H–NMR spectra in the fluorinated ethylene propylene copolymer (FEP) insert at the temperature of 80 °C. The gas-phase signals are highlighted by red color. The spectra were not referenced.

To eliminate the influence of the glass surface on the observed data, we decided to utilize a different material for the NMR tube insert. There are commercially available tube liners made of FEP, which is a highly inert material. Unfortunately, these inserts are not airtight, which causes complications when they are used to measure gas samples. However, we exploited the slow gas leakage for the measurement of the pressure dependence of NMR spectra, where pressure is a function of time. The inserts were filled with a few drops of liquid sample and subsequently heated up to the desired temperature. Figure 4 shows an example of the time and thus indirect pressure dependence of the ^1^H–NMR spectra of acetic acid. The signal broadening was no longer present in the spectra, which suggested that this was, indeed, a glass-specific effect. This fact also provided a possible explanation for the inaccuracy of the resultant values of ∆*H* and ∆*S* in the previous NMR study discussed in the introduction, in which glass NMR tubes were used [8].

**Figure 5:**
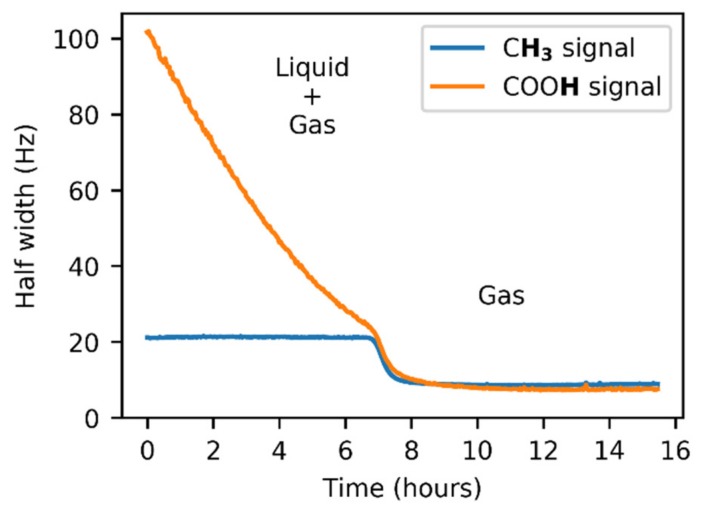
The ^1^H–NMR line broadening in the presence of acetic acid vapors and condensate. A transition is observed after the liquid evaporation.

The signal intensities varied during the initial phase of the experiment in the FEP insert due to the line shape broadening caused by the exchange between the liquid and gas states. It is evident from Figure 5 that the line broadening was exhibited by both acetic acid signals. Whereas the COOH linewidth showed a strong dependence on time, the signal of the methyl group tended to have a constant width, with the only disruption during the *l* + *g* → *g* transition.

During the measurements in the FEP inserts, another set of acetic acid signals was also present in the spectra. These, however, did not come from the inside of the FEP insert; rather, they came from the small space between the insert and the enclosing NMR tube. The vapors escaping from the insert tended to condensate there; therefore, the signals corresponded to liquid acetic acid. The signals of the gas-state molecules inside the insert were thus not affected by those “outer” molecules.

**Figure 6:**
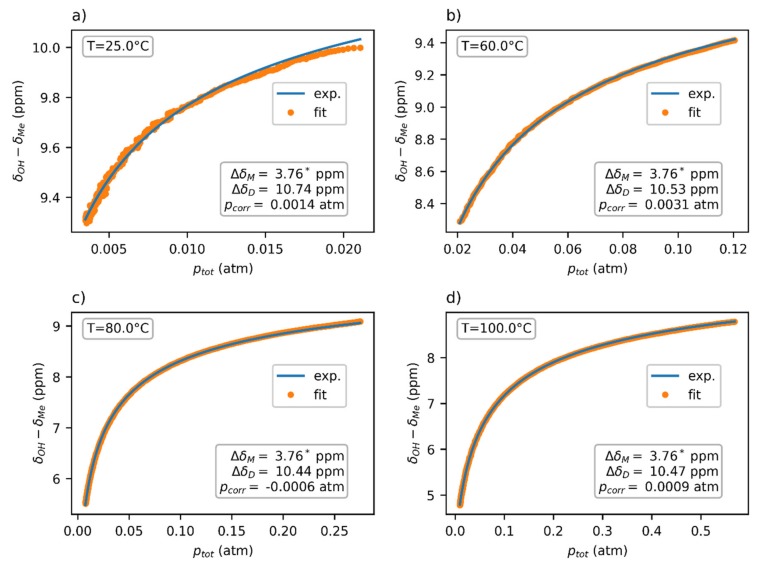
The pressure dependence of the chemical shift of acetic acid vapors in the FEP insert fitted using Expression (2). The chemical shift ∆*δ_M_* was obtained independently through extrapolation from a 150 °C dataset. Note that the *p*_tot_ values used in the fit were based on the experimentally obtained concentration. The experimental temperatures were (**a**) 25 °C, (**b**) 60 °C, (**c**) 80 °C and (**d**) 100 °C.

The thermodynamic parameters ∆*H* and ∆*S* were globally fitted to four datasets at temperatures 25*,* 60*,* 80, and 100 °C (Figure 6), yielding the values ∆*H* = (−15.4 ± 0.5) kcal*/*mol, ∆*S* = (−36.6 ± 1.5) cal*/*mol·K, and ∆*G*^0^ = (−4.48 ± 0.12) kcal*/*mol. The chemical-shift difference *∆δ* of the dimer was separately determined for each dataset, with the average value being ∆*δ_D_* = 10.55 ppm. The chemical shift of the monomer ∆*δ_M_* = 3.76 ppm was obtained through the extrapolation of the chemical-shift dependence at 150 °C to zero pressure.

A visual analysis of the fitted data (Figure 6) showed a worse fit at lower temperatures, especially at 25 °C. This could have partly been caused by a higher noise in the data at lower temperatures, which is also apparent in the picture. However, the possibility of another chemical exchange between other supramolecular structures cannot be excluded. Since these supramolecular forms (e.g., trimers and tetramers) would be entropically less stable than dimers, they would only exist in the acetic acid vapors at low temperature and/or high pressure. This hypothesis would explain why our model, which considers only monomers and dimers, fit better to the high-temperature data.

### 3.2. Computations

Three different computational methods were employed for the calculation of the binding energy of the acetic acid dimer. B3LYP is a widely used DFT functional due to its speed and good results for organic molecules. The second-order Møller–Plesset perturbation theory was chosen as a popular representative of post-Hartree–Fock methods. MP2 scaled sensibly with the system size and allowed for the geometry optimization of the acetic acid dimer with a quadruple-*ζ* basis set. The quite demanding coupled-cluster method with single, double excitations, and triple excitations as a perturbation CCSD(T) is one of the most precise methods for electronic-energy calculations today.

#### 3.2.1. The Structure of the Acetic Acid Dimer

Geometry optimization was carried out using DFT and MP2 with the Aug-cc-pVQZ basis set. In the DFT case, the B3LYP functional was used both with and without Grimme’s empirical dispersion corrections (GD3). Table 1 summarizes selected inter-atomic distances in the optimized structures of the acetic acid dimer. The dispersion correction added slightly more cohesion to the system. This affected the interatomic distance between the oxygens involved in hydrogen bonding (O···H–O), where the shortening of 0.007 Å was observed when compared to the dispersion-uncorrected B3LYP. The distance shortening of 0.01 Å was also observed between the carboxylic carbons, which was related to the valence-angle change of the carboxylic group. There was no significant change of the C–O and C–C bond lengths. Generally, dispersion correction seemed to change the B3LYP geometry more towards the MP2-optimized structure. The atomic coordinates of these structures can be found in the Supplementary Materials.

#### 3.2.2. Dimerization Energy

Thermodynamic contributions to the ∆*H* and ∆*S* of dimerization were obtained from thermochemical calculations using the Aug-cc-pVTZ basis set. These values were added to the single-point energies in order to obtain the standard Gibbs energy ∆*G*^0^ (at *T* = 298 K and *p* = 1 atm), the enthalpy ∆*H*, and entropy ∆*S* of the dimer formation, which are directly measurable and were thus better suited for the comparison with the experiments than the change of electronic energy ∆*E*.

Table 2 shows that the B3LYP functional without dispersion corrections systematically underestimated the binding energy in comparison with MP2 and CCSD(T) approaches. On the other hand, the use of dispersion-corrected B3LYP led to the value of dimerization energy being more than 1 kcal/mol higher than the experimentally observed value. Post-HF methods, MP2 and CCSD(T), gave much more accurate energies, with deviations from the experimental values of enthalpy and Gibbs energy lower than 0.2 kcal*/*mol at the CBS limit. One should keep in mind the experimental errors of 0.1 kcal*/*mol for Gibbs energy and 0.5 kcal*/*mol and 1.5 cal*/*mol·K for enthalpy and entropy, respectively.

The basis set superposition error: The binding energies in Table 2 were not corrected for the BSSE. Table S4 shows BSSE estimates based on the counterpoise method. B3LYP computations with the Aug-cc-pVQZ basis set exhibited a relatively small BSSE (0.13 kcal/mol), regardless of whether the dispersion correction was applied or not. Even after the BSSE correction, the B3LYP functional provided an inaccurate prediction of acetic acid binding energies.

Like in the case of B3LYP, the BSSE was estimated by the counterpoise method on the MP2/Aug-cc-pVQZ structure. As arises from Table S4, the BSSE was nearly six times higher (0.87 kcal/mol) in the case of MP2 than in the case of B3LYP with the identical basis set. The application of these corrections led to lower binding energy than the CBS extrapolation. In the case of MP2, even the BSSE-uncorrected binding energy was closer to the CBS value than the counterpoise-corrected one. This is not uncommon; it is known that the counterpoise method systematically overestimates the BSSE [52]. Extensive studies of this subject suggest that there is an error cancellation between the BSSE and the basis set completeness error (BCE), as a result of which the compensation of the BSSE may cause the worsening of the binding-energy value due to the effective decompensation of the BCE [25,53]. It has previously been observed that the average of BSSE-corrected and uncorrected values yields better agreement with the calculations at the CBS limit [54]. Our calculations confirmed this observation; taking only one half of the BSSE improved the result significantly (Table S4).

NMR shielding calculation and PIMD simulations: The calculated ^1^H–NMR chemical-shift differences *δ_OH_–δ_Me_* of the acetic acid monomer and dimer are summarized in Table 3. In the case of the monomer, both the MP2 and B3LYP methods performed similarly. The deviation from the experimental value was between 0.1 and 0.25 ppm, where B3LYP seemed to provide slightly better results. These small deviations can be explained by vibrational effects, which are discussed below. A quite different situation occurred in the case of the dimer. Though the B3LYP- and MP2-calculated chemical shifts were nearly the same (with the differences being smaller than 0.15 ppm), the calculated value was more than 1 ppm higher than the experimental value. This suggests that the deviation from the experimental value probably did not originate in the inaccuracy of the methods used.

We hypothesize that the large difference between the experimental and calculated values of Δ*δ*_D_ could have originated from the lack of vibrational averaging in the calculations. In the case of the monomer, the vibrational corrections to both the COOH and methyl protons have been found to be similar in several cases [55] and thus might largely have cancelled out in the reported Δ*δ*_M_ value. However, the vibrational correction of the carboxylic hydrogen might have been significantly higher in the dimeric case because of the more prominent potential anharmonicity than in the case of the monomer.

In order to estimate the importance of the vibrational-averaging contribution to the chemical shifts, we performed PIMD simulations of the monomer and dimer of acetic acid. This method was chosen for its ability to comprehend nuclear quantum effects such as quantum nuclear delocalization and tunneling, exhibited especially by low-mass atoms like hydrogen. It has previously been demonstrated that PIMD simulations combined with DFT shielding calculations provide excellent agreement with experimental solid-state NMR chemical-shift changes induced by temperature change or by deuterium isotope substitution, which are the consequences of vibrational averaging [56,57]. However, these calculations also revealed that the PBE functional used in these simulations underestimated the energetic barriers of proton transfer [58]. Furthermore, the PIMD simulations and subsequent shielding calculations are computationally very demanding because a large number (hundreds to thousands) of geometry snapshots must be used to obtain sufficiently converged results.

As can be seen in Table 3, the PIMD-based vibrational correction to the monomer Δ*δ_M_* value was small and negative, and when added to the B3LYP or MP2 values, the agreement with experiment was improved. However, in the case of the dimer, the shielding calculations did not converge, even after averaging 1000 geometry snapshots from the PIMD simulation. This poor convergence led to differences between the shieldings of the equivalent OH protons in the dimer. Furthermore, the probability distributions of the calculated Δ*δ_D_* values were very broad (Figure S9), and insufficient convergence could thus have led to large errors in the calculated Δ*δ_D_* values. We also noticed that the use of the PBE functional in the PIMD simulations led to the oversampling of the dimer geometries, which were characterized by high values of the chemical-shift difference Δ*δ_D_* (Figure S10). Therefore, the values of the vibrational correction for the dimer were probably biased towards positive values and unreliable.

## 4. Conclusions

We performed gas-phase NMR measurements of acetic acid at variable pressures and temperatures. These experiments allowed us to extract the thermodynamic parameters of acetic acid dimerization. These values were in good agreement with previous values based on IR experiments and vapor-density or thermal-conductivity measurements. We noticed, however, that the gas-phase measurements of acetic acid had to be performed in inert polymer tubes because interactions of acetic acid with the surface of glass tubes led to significantly biased results. These experiments also provided chemical shifts of isolated monomeric and dimeric forms of acetic acid. 

The temperature and pressure dependence of the observed chemical shifts of acetic acid was in very good agreement with the dependences derived for the basic dimerization model. We only observed minor deviations for the lowest experimental temperature (25 °C). These deviations may indicate the presence of other supramolecular form(s), such as those of the trimer or tetramer, of acetic acid at low temperatures in the gas phase. 

The experimental thermodynamic parameters and chemical shifts were compared with those obtained by DFT, MP2, and CCSD(T) calculations. The calculated thermodynamic parameters of the dimerization and the chemical-shift difference *δ_OH_–δ_Me_* of the acetic acid monomer were in very good agreement with the experimental values. However, the calculated chemical-shift difference *δ_OH_–δ_Me_* of the acetic acid dimer was ca 1 ppm higher than the experimental value. We hypothesize that this overestimation was caused by neglecting the vibrational averaging in the calculations. We intended to estimate the vibrational contribution to the chemical shifts by PIMD simulations and subsequent shielding calculations for geometry snapshots. Unfortunately, we found out that the computational method used in the PIMD simulations (the PBE functional) was not suitable for this task because it oversampled the geometries with very short OH∙∙∙O hydrogen-bond distances. However, these results demonstrate the importance of an accurate treatment of vibrational corrections to NMR parameters.

## Figures and Tables

**Table 1:** Interatomic distances (Å) and the O–C–O valence angle (∢, °) in the cyclic dimer of acetic acid obtained at the second order Møller–Plesset perturbation theory (MP2) level and their differences from the values obtained at the B3LYP level. GD3: Grimme’s dispersion correction.

Distance/Angle	MP2	B3LYP	B3LYP and GD3
O–H	0.998	0.002	0.003
O–H···O	2.650	0.012	0.005
C1,C1’	3.832	0.015	0.005
C1–C2	1.495	0.006	0.006
C=O	1.226	−0.003	−0.003
C–O	1.319	0.000	−0.001
∢ O–C–O	123.90	−0.16	0.00

**Table 2:** The calculated values of the dimerization enthalpy (kcal/mol), entropy (cal/mol·K), and Gibbs energy (kcal/mol) of acetic acid at different computational levels. The results for the MP2 and CCSD(T) (coupled cluster with single, double and perturbative triple excitation) methods rely on MP2/Aug-cc-pVTZ thermochemical calculation. Aug-cc-pVDZ: augmented correlation-consistent double-*ζ*; V5Z: quintuple-*ζ*; CBS: complete basis set.

Method	Basis Set	∆*H*	∆*S*	∆*G*^0^
B3LYP	Aug-cc-pVQZ	−14.27	−37.43	−3.11
B3LYP and GD3	Aug-cc-pVQZ	−16.77	−37.25	−5.67
MP2	Aug-cc-pVTZ	−15.93	−37.12	−4.86
MP2	Aug-cc-pVQZ	−15.57 *		−4.51 *
MP2	Aug-cc-pV5Z	−15.34 *		−4.28 *
MP2	CBS ^[a]^	−15.31 *		−4.24 *
CCSD(T)	Aug-cc-pVDZ	−15.83 *		−4.76 *
CCSD(T)	Aug-cc-pVTZ	−16.08 *		−5.02 *
CCSD(T)	CBS ^[b]^	−15.46 *		−4.40 *
exptl.		−15.38	−36.6	−4.48

[a] The complete basis set limit according to Equation (3); [b] The MP2-based complete basis set limit according to Equation (4). The asterisks indicate enthalpy and Gibbs energy values, for which different computational levels for electronic energy and thermochemistry were used.

**Table 3:** The calculated ^1^H–NMR chemical-shift differences *δ_OH_–δ_Me_* (ppm) of the acetic acid monomer and dimer. The computations were performed on the structures optimized with the Aug-cc-pVQZ basis set using the same method as the subsequent NMR calculation.

Method	Monomer	Dimer
MP2	4.01	11.77
B3LYP	3.90	11.81
B3LYP and GD3	3.86	11.91
Vibrational correction ^[a]^	−0.08	−0.05
Experiment	3.76	10.55

[a] Calculations based on path-integral molecular dynamics (PIMD) simulations and the averaging of NMR parameters calculated for 1000 geometry snapshots.

## Supplementary Materials

The following are available online at https://www.mdpi.com/1420-3049/25/9/2150/s1. Detailed derivation of expressions used in the data fitting, Antoine equations for vapor pressure of acetic acid, and estimation of the water content in the acetic acid samples, Figure S1: ^1^H–NMR spectra at –6 °C of a mixture of acetic acid and water, which remains after freezing of pure acetic acid out of the solution. Figure S2: Temperature dependence of chemical shift of acetic acid at various concentrations. Table S1: Optimized thermodynamic parameters of dimerization and chemical shifts of monomer and dimer from measurements of acetic acid in the glass insert. Figure S3: Temperature dependence of the half width of the ^1^H–NMR signal of acetic acid COOH group in the glass insert. Figure S4: Temperature series of ^1^H–NMR spectra of acetic acid (c = 1.1 mM) in the glass insert. Figure S5: Temperature series of ^1^H–NMR spectra of acetic acid (c = 4.4 mM) in the glass insert. Figure S6: Temperature series of ^1^H–NMR spectra of acetic acid (c = 7.9 mM) in the glass insert. Figure S7: Pressure dependence of the chemical shift difference of acetic acid in the FEP insert. Table S2: Optimized thermodynamic parameters of dimerization and the chemical shift difference of the dimer obtained from the measurements of acetic acid in the FEP insert. Figure S8: Complete basis set limit of correlation energy of acetic acid monomer and dimer. Table S3: Computed energy values used for the CBS extrapolation. Table S4: Calculated values of dimerization energies of acetic acid at different computational levels. Table S5: Cartesian coordinates of the acetic acid monomer optimized at the MP2/Aug-cc-pVQZ level. Table S6: Cartesian coordinates of the acetic acid dimer optimized at the MP2/Aug-cc-pVQZ level. Figure S9: Distribution of chemical shifts of acetic acid monomer and dimer (bottom) during the PIMD simulation. Figure S10: Correlation between B3LYP calculated chemical shift and O-H distance in the dimer of acetic acid. Table S7: B3LYP calculated ^1^H chemical shieldings (ppm) and vibrational corrections based on the PIMD geometries.
