# Supplementary material for: Dimerization of Acetic Acid in the Gas Phase—NMR Experiments and Quantum-Chemical Calculations
Source: Molecules. 2020 May 4;25(9):2150. doi: 10.3390/molecules25092150 (PMC7248931; doi:10.3390/molecules25092150)
Supplement: Supplementary file 1 [file molecules-25-02150-s001.pdf]

Supporting Information: Dimerization of acetic acid in the gas phase – NMR experiments and quantum-chemical calculations

Ondřej Socha, Martin Dračínský

Institute of Organic Chemistry and Biochemistry, AS CR, Flemingovo 2, Prague, Czech Republic. E-mail: dracinsky@uochb.cas.cz

Contents

1 Detailed derivation of expressions used in the data fitting **2**

2 Antoine equations for vapor pressure of acetic acid **3**

3 Estimation of the water content in the acetic acid smaples **3**

4 Glass dataset fitting **4**

5 FEP dataset fitting **9**

6 Quantum chemical computations **10**

7 Atomic coordinates of monomer and dimer of acetic acid. **12**

8 Results of the path integral molecular dynamics (PIMD) **13**

1 Detailed derivation of expressions used in the data fitting

The dimerization is assumed to be a chemical exchange between the monomeric M and dimeric D form

$$2M \rightleftharpoons D.$$

The equilibrium constant K is then defined as

$$K = \frac{p_D}{p_M^2}. \tag{S1}$$

The total pressure can be expressed as a sum of partial pressures of the monomer p_M and the dimer p_D (Dalton law)

$$p_{tot} = p_M + 2p_D. \tag{S2}$$

From expression (S1)

$$Kp_M^2 = p_D$$

considering formula (S2) leads to quadratic equation

$$2Kp_M^2 = p_{tot} - p_M \tag{S3}$$

with discriminant

$$D = 1 - 8Kp_{tot}.$$

There are two solutions of eq. (S3)

$$p_M = \frac{\pm\sqrt{D} - 1}{4K}.$$

Discarding the negative root, we obtain the final expression for the partial pressure of monomers

$$p_M = \frac{\sqrt{1 - 8Kp_{tot}} - 1}{4K},$$

where equilibrium constant K can be expressed using thermodynamic parameters

$$K = \exp\left(-\frac{\Delta G}{RT}\right) = \exp\left(-\frac{\Delta H - T\Delta S}{RT}\right).$$

Fast chemical exchange between the monomer M and dimer D leads to averaged NMR signals, whose chemical shift δ is calculated as weighted average of the chemical shifts of the monomer δ_M and the dimer δ_D

$$\delta = \frac{p_M\delta_M + 2p_D\delta_D}{p_{tot}}.$$

p_D can be substituted using eqation (S2)

$$\delta = \frac{p_M\delta_M + (p_{tot} - p_M)\delta_D}{p_{tot}}.$$

After final rearrangement

$$\delta = \frac{p_M}{p_{tot}}(\delta_M - \delta_D) + \delta_D, \tag{S4}$$

where $p_M = p_M(T, \Delta H, \Delta S)$ is a function of the thermodynamic parameters, $p_{tot} = p_{tot}(T, c)$ depends on temperature and concentration. Values of δ_M, δ_D are considered to be independent. The observed chemical shift is thus a function of the mentioned parameters

$$\delta = \delta(T, c, \Delta H, \Delta S, \delta_M, \delta_D).$$

2 Antoine equations for vapor pressure of acetic acid

The following formulae [1] were used to calculate vapor pressure p of acetic acid at given temperature T.

$$p_{mmHg} = 10^{7.80307 - \frac{1651.2}{225+T}} \text{ for } 0°\text{C} < \text{T} \leq 36°\text{C}$$

$$p_{mmHg} = 10^{7.18807 - \frac{1416.7}{211+T}} \text{ for } 36°\text{C} < \text{T} \leq 170°\text{C}$$

Temperature T is in units of °C. The pressure was converted from units of mmHg to atm using expression

$$p_{atm} = 0.001316 * p_{mmHg}$$

3 Estimation of the water content in the acetic acid smaples

Acetic acid used for sample preparation in our experiments was delivered from the manufacturer in 1.5 ml glass ampules. Water content was determined using standard liquid NMR measurement based on the following: Pure acetic acid solidifies from the solution (acetic acid – water) at temperatures under the melting temperature of acetic acid. However, a mixture of remaining acetic acid and water stays liquid above −27°C, which is the eutectic point of the mixture. An NMR tube filled with 0.5 ml of acetic acid was cooled down below the freezing point of acetic acid. In Figure S1, two NMR spectra at −6°C are shown. One spectrum corresponds to the acetic acid directly from the ampule and the other one is with the addition of 7 mol% of water. The left signal belongs to the COOH hydrogen, which is exchanging with the water, whereas the right signal is from the methyl group of acetic acid. It is clear from the comparison of these two spectra, that there is more than ten times less water in the original sample. Therefore, we conclude, that the water content in the acetic acid used in our experiments is far below 1 mol%.

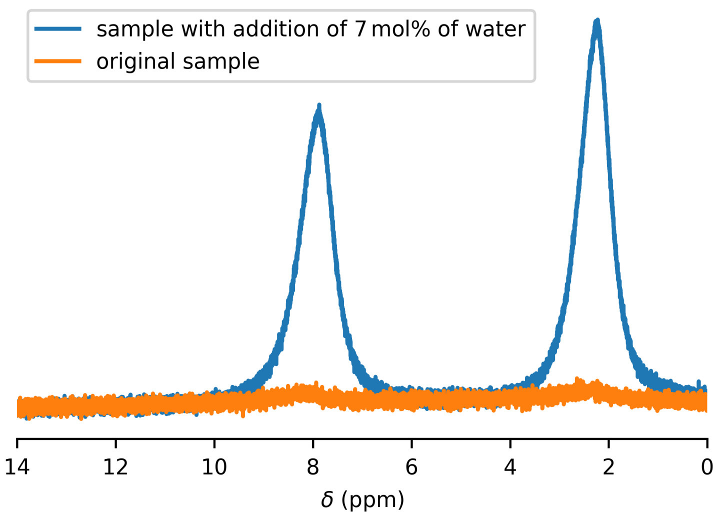

Figure S1: ^{1}H NMR spectra at −6°C of a mixture of acetic acid and water, which remains after freezing of pure acetic acid out of the solution. Single scan with 0.5 Hz exponential apodization.

4 Glass dataset fitting

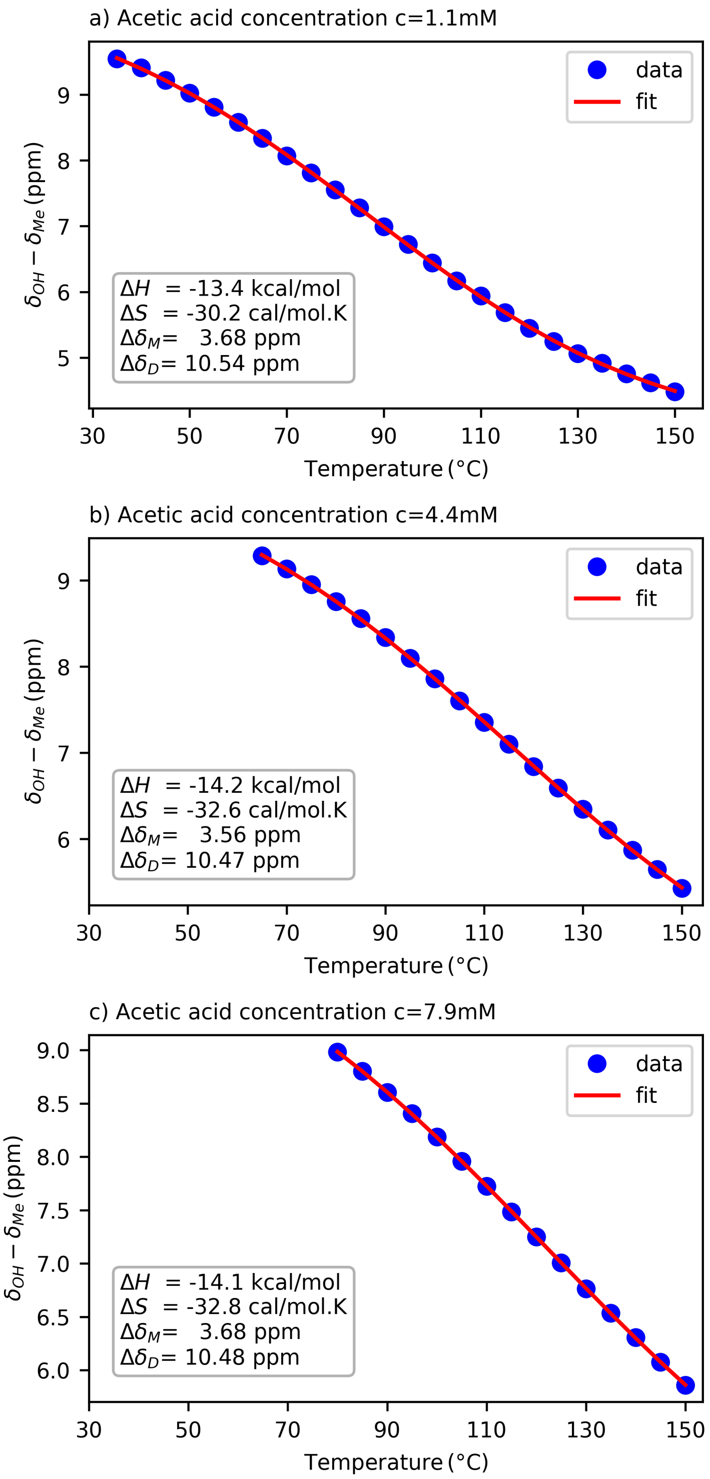

Figure S2: Temperature dependence of chemical shift difference of acetic acid in the glass insert at various concentrations. Fitted using expression (S4).

Table S1: Optimized thermodynamic parameters of dimerization and chemical shifts of monomer and dimer from measurements of acetic acid in the glass insert. The value T_0 corresponds to the condensation temperature and p_0 is the pressure at this temperature.

T_0	p_0	c	ΔH	ΔS	$\Delta\delta_M$	$\Delta\delta_D$
°C	atm	mM	kcal/mol	cal/mol·K	ppm	ppm
30	0.0280	1.13	-13.4±0.1	-30.3±0.4	3.68±0.03	10.54±0.03
60	0.1201	4.39	-14.2±0.2	-32.6±0.7	3.56±0.09	10.47±0.04
75	0.2258	7.90	-14.1±0.3	-32.8±0.8	3.68±0.12	10.48±0.05

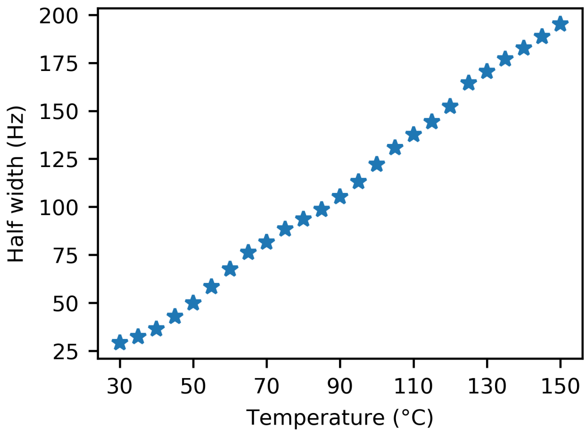

Figure S3: Temperature dependence of the half width of the ^{1}H NMR signal of acetic acid COOH group in the glass insert.

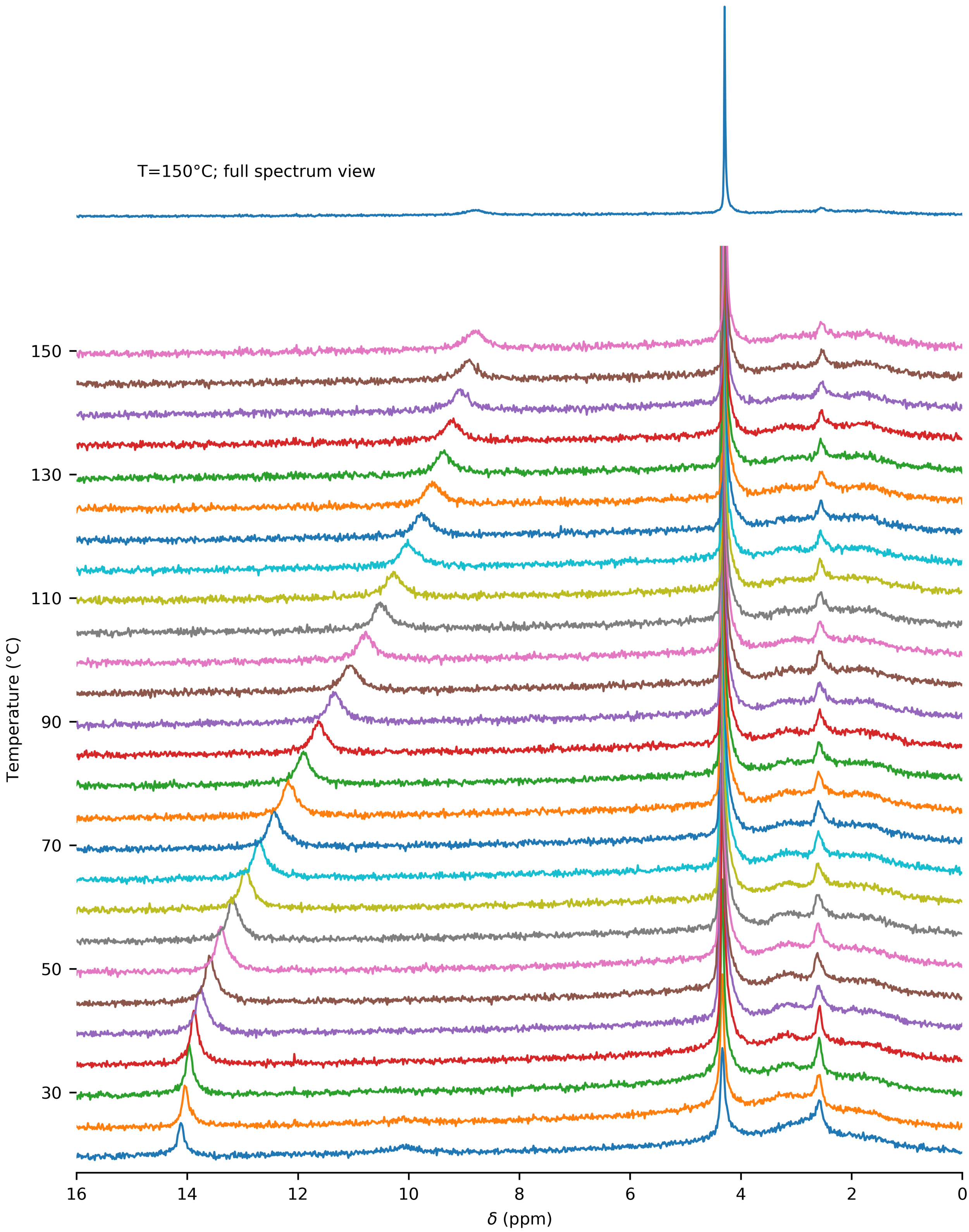

Figure S4: Temperature series of ^{1}H NMR spectra of acetic acid (c = 1.1 mM) in the glass insert. The spectra were not referenced.

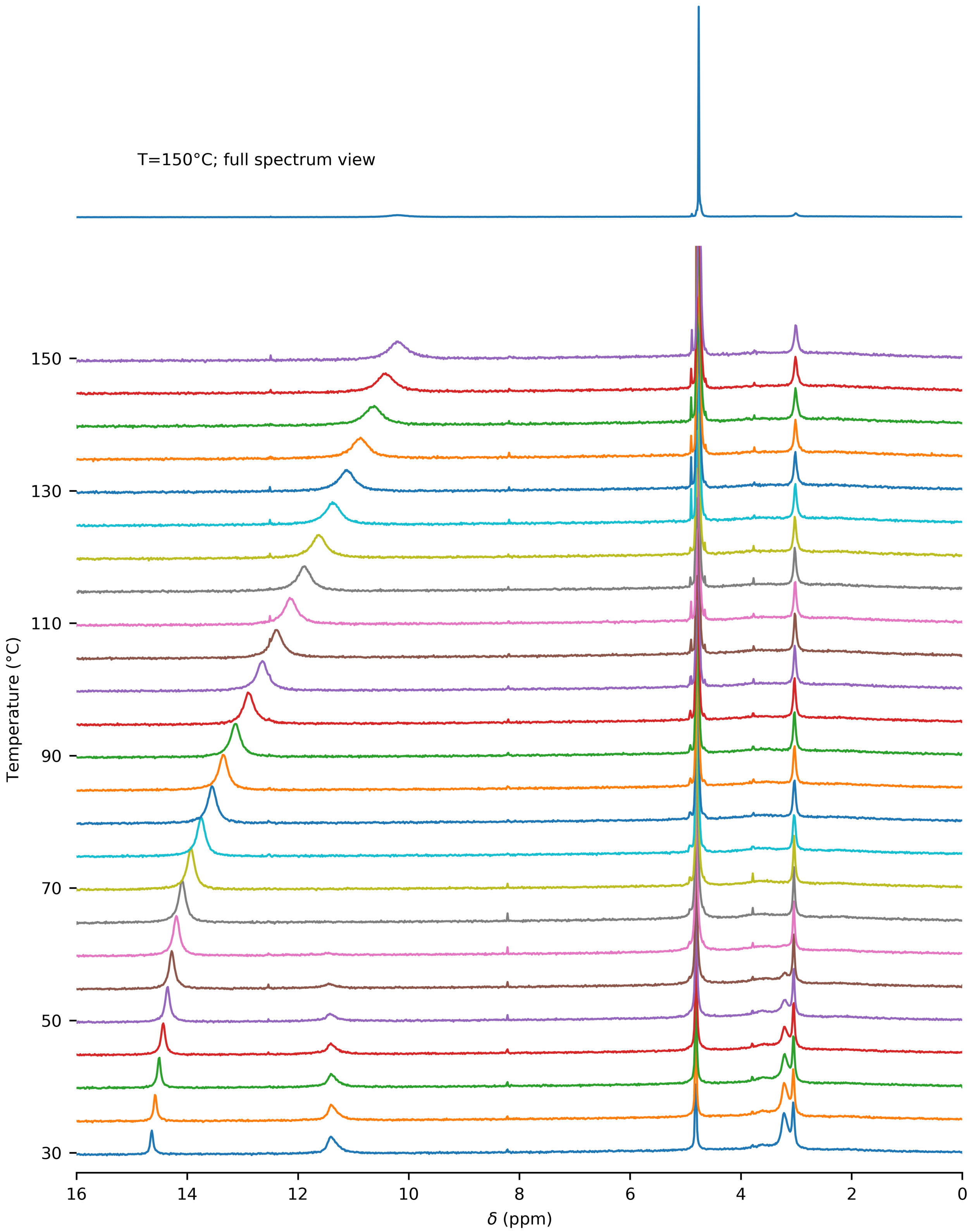

Figure S5: Temperature series of ^{1}H NMR spectra of acetic acid (c = 4.4 mM) in the glass insert. The spectra were not referenced.

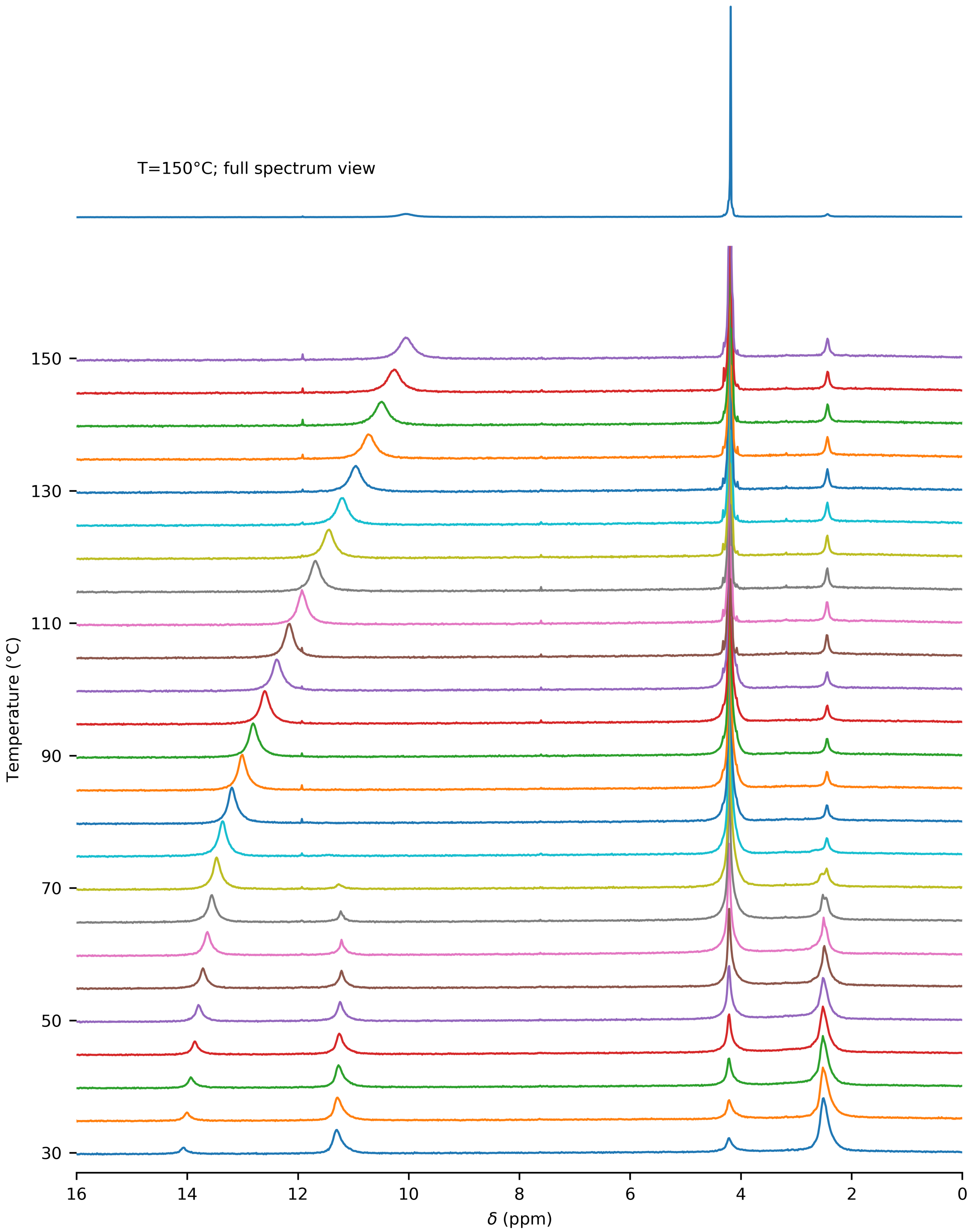

Figure S6: Temperature series of ^{1}H NMR spectra of acetic acid (c = 7.9 mM) in the glass insert. The spectra were not referenced.

5 FEP dataset fitting

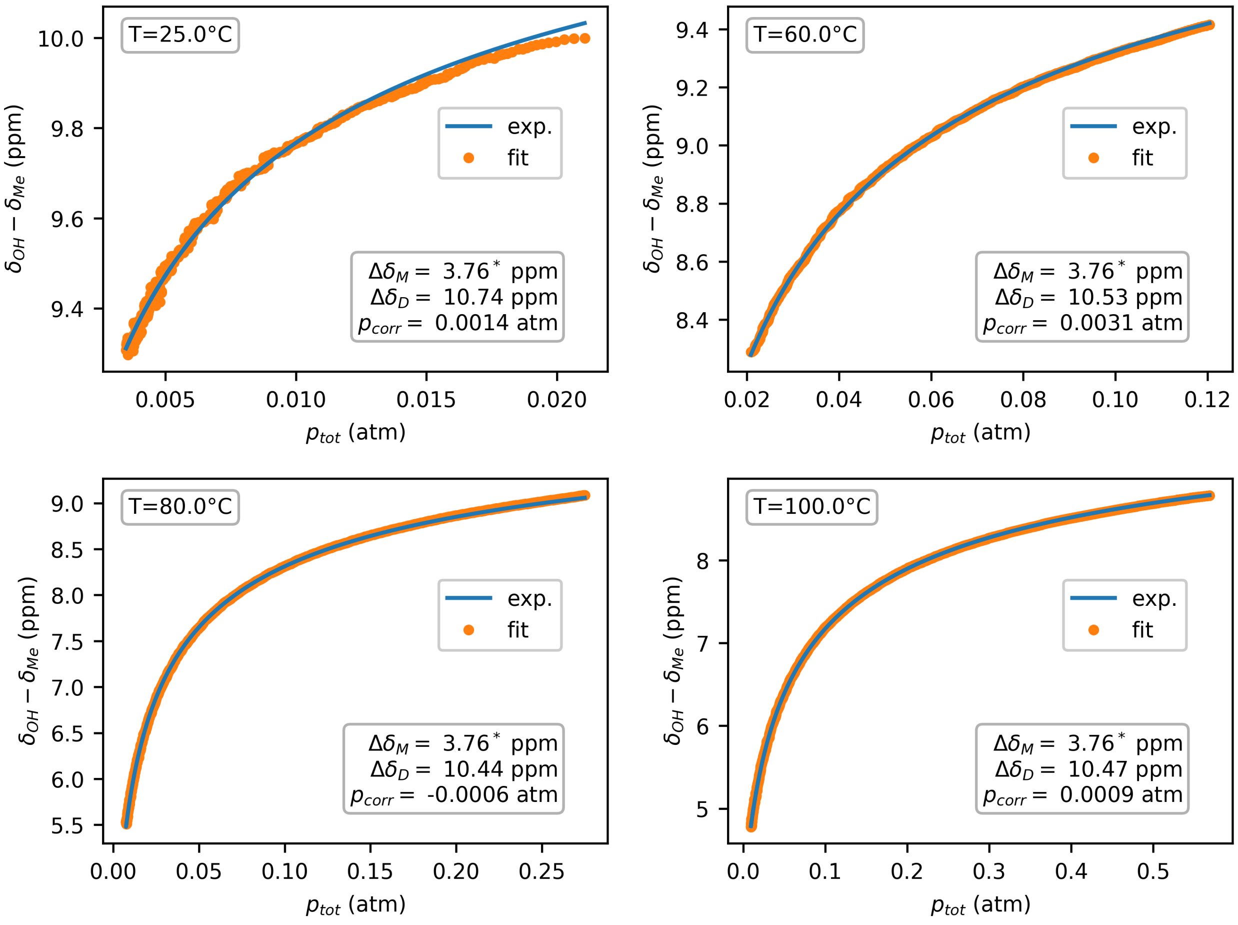

Figure S7: Pressure dependence of the chemical shift difference of acetic acid in the FEP insert. The chemical shift difference $\Delta\delta_M$ was obtained independently trough extrapolation from 150°C dataset. Note that the p_{tot} values are based on the experimentally obtained concentration.

Table S2: Optimized thermodynamic parameters of dimerization and the chemical shift difference of the dimer obtained from the measurements of acetic acid in the FEP insert. The chemical shift difference of the monomer was fixed during the optimization.

T (°C)	$\Delta\delta_D$ (ppm)	p_{corr} (atm)	ΔH (kcal/mol)	ΔS (cal/mol·K)	ΔG^0 (kcal/mol)
25	10.74±0.06	0.0014±0.0005	-15.38±0.54	-36.6±1.5	-4.48±0.12
60	10.53±0.05	0.0031±0.0011			
80	10.44±0.03	-0.0006±0.0002			
100	10.47±0.02	0.0009±0.0001			

$\mathrm{Cov}(\Delta H, \Delta S) = 0.80 \cdot 10^{-3}$ kcal^2/mol^2·K
ΔG^0 at 25°C and 1 atm

6 Quantum chemical computations

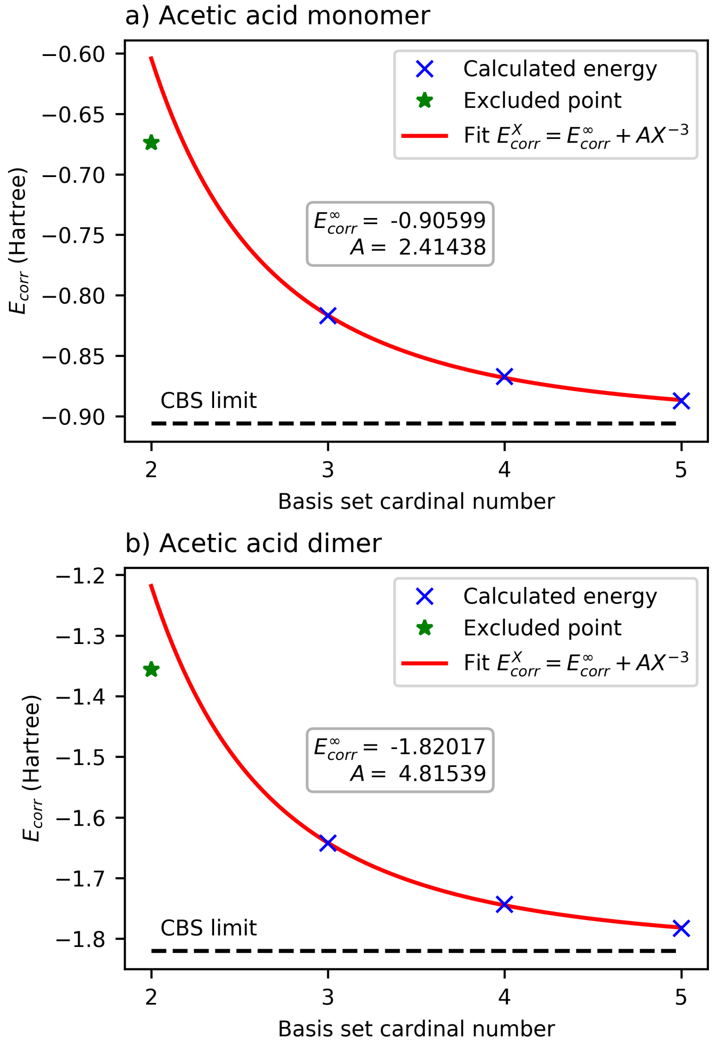

Figure S8: Complete basis set limit of correlation energy of acetic acid monomer (a) and dimer (b).

Table S3: Computed energy values used for the CBS extrapolation. Hartree-Fock energies in Aug-cc-pV5Z basis set were taken as converged.

X	Monomer (Hartree)		Dimer (Hartree)		Binding energy (kcal/mol)		
	E^{HF}	E^{MP2}_{corr}	E^{HF}	E^{MP2}_{corr}	E^{HF}	E^{MP2}_{corr}	E^{MP2}
2	-227.849493	-0.673919	-455.718203	-1.356092	-12.06	-5.18	-17.24
3	-227.905765	-0.816810	-455.830619	-1.642292	-11.98	-5.44	-17.42
4	-227.920441	-0.867330	-455.859602	-1.743135	-11.75	-5.32	-17.06
5	-227.923855	-0.887359	-455.866287	-1.782968	-11.66	-5.18	-16.83
CBS		-0.905987		-1.820169		-5.14	-16.80

Table S4: Calculated values of dimerization energies of acetic acid at different computational levels. All energy values are expressed in units of kcal/mol, entropy in cal/mol·K. Zero point energy (ZPE) is included in the thermal enthalpy $\Delta H_{thermal}$.

Method	Basis set	ΔE	$\Delta H_{thermal}$	ΔH	ΔS	ΔG^0	BSSE
B3LYP	Aug-cc-pVQZ	-15.57	1.30	-14.27	-37.43	-3.11	0.13
B3LYP+GD3	Aug-cc-pVQZ	-18.11	1.34	-16.77	-37.25	-5.67	0.13
MP2	Aug-cc-pVTZ	-17.42	1.49	-15.93	-37.12	-4.86	
MP2	Aug-cc-pVQZ	-17.06		-15.57		-4.51	0.87
MP2	Aug-cc-pV5Z	-16.83		-15.34		-4.28	
MP2	CBS	-16.80		-15.31		-4.24	
CCSD(T)	Aug-cc-pVDZ	-17.32		-15.83		-4.76	
CCSD(T)	Aug-cc-pVTZ	-17.57		-16.08		-5.02	
CCSD(T)	CBS	-16.95		-15.46		-4.40	
exptl.	n/a	-	-	-15.38	-36.55	-4.48	n/a

7 Atomic coordinates of monomer and dimer of acetic acid.

Table S5: Cartesian coordinates of the acetic acid monomer optimized at MP2/Aug-cc-pVQZ level.

At.	X (Å)	Y (Å)	Z (Å)
C	-3.393376	0.885977	0.058367
C	-1.899265	0.918210	0.111216
H	-3.774074	1.894956	-0.031793
H	-3.778471	0.416597	0.959335
H	-3.713825	0.284852	-0.787989
O	-1.383043	-0.328549	0.224316
H	-0.422413	-0.215918	0.251366
O	-1.204908	1.904314	0.062557

Table S6: Cartesian coordinates of the acetic acid dimer optimized at MP2/Aug-cc-pVQZ level.

At.	X (Å)	Y (Å)	Z (Å)
C	-3.421233	0.885771	0.077200
C	-1.929684	0.984427	0.093432
H	-3.854359	1.864401	-0.084568
H	-3.763566	0.471368	1.021769
H	-3.726867	0.201383	-0.709776
O	-1.338463	-0.178227	0.287243
H	-0.348662	-0.051744	0.288502
O	-1.326976	2.041065	-0.058536
C	1.896997	1.176006	0.144657
C	3.388546	1.274661	0.160878
H	3.821673	0.296029	0.322634
H	3.694185	1.959039	0.947862
H	3.730876	1.689076	-0.783687
O	1.305776	2.338662	-0.049142
H	0.315975	2.212180	-0.050393
O	1.294289	0.119370	0.296634

8 Results of the path integral molecular dynamics (PIMD)

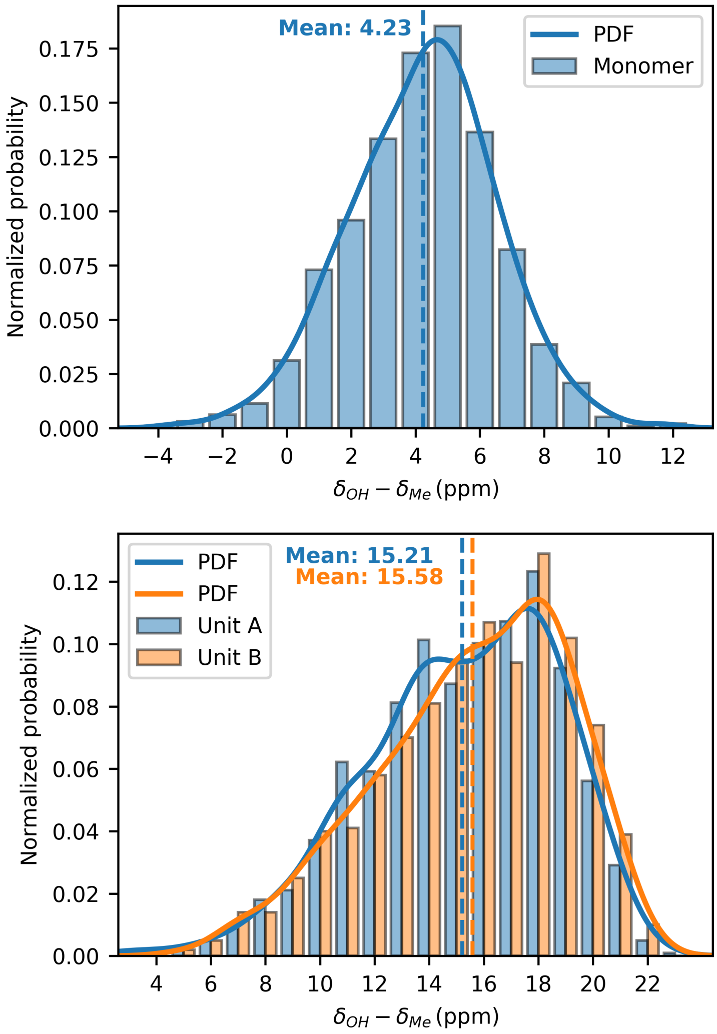

Figure S9: Distribution of chemical shifts of acetic acid monomer (top) and dimer (bottom) during the PIMD simulation. Probability density function (PDF) was estimated using gaussian kernel density estimation.

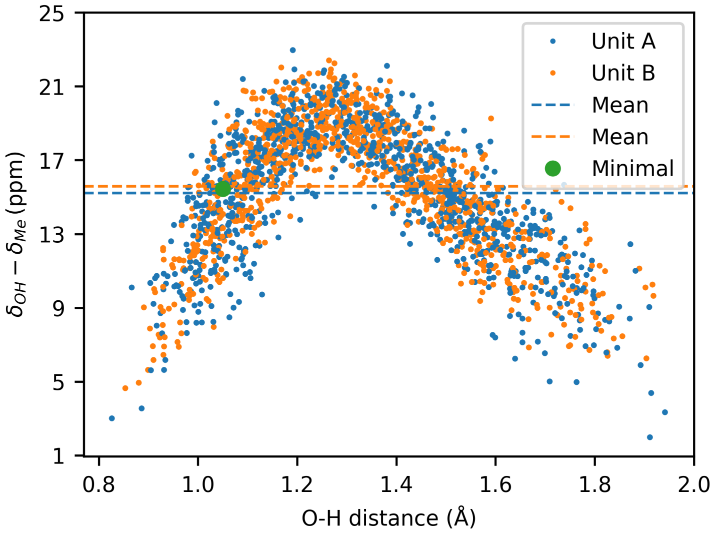

Figure S10: Correlation between B3LYP calculated chemical shift and O-H distance in the dimer of acetic acid. Data are based on the PIMD simulation employing the PBE functional. The green dot indicates the PBE optimized structure.

Table S7: B3LYP calculated ^{1}H chemical shideldings (ppm) and vibrational corrections, based on the PIMD geometries. The optimized structure was obtained at the same computational level as the PIMD simulation.

	Monomer			Dimer		
	σ_{Me}	σ_{OH}	$\sigma_{Me} - \sigma_{OH}$	σ_{Me}	σ_{OH}	$\sigma_{Me} - \sigma_{OH}$
Optimized strucutre	29.31	24.99	4.31	29.27	13.82	15.45
PIMD average	28.48	24.25	4.23	28.64	13.25	15.40
Vibrational correction	-0.83	-0.75	-0.08	-0.63	-0.58	-0.05

References

[1] N. de Nevers, *Air Pollution Control Engineering: Second Edition*, Waveland Press, **2010**, p. 586.
